# Impact of prior abdominal surgery on the outcomes after robotic - assisted laparoscopic radical prostatectomy: single center experience

**DOI:** 10.1590/S1677-5538.IBJU.2015.0607

**Published:** 2016

**Authors:** Nozomu Kishimoto, Tetsuya Takao, Gaku Yamamichi, Takuya Okusa, Ayumu Taniguchi, Koichi Tsutahara, Go Tanigawa, Seiji Yamaguchi

**Affiliations:** 1 Osaka General Medical Center-Urology, Osaka, Japan

**Keywords:** Surgical Procedures, Operative, Robotic Surgical Procedures, Laparoscopy

## Abstract

**Purpose:**

To evaluate the influence of prior abdominal surgery on the outcomes after robotic-assisted laparoscopic radical prostatectomy (RALP).

**Materials and Methods:**

We retrospectively analyzed patients with prostate cancer who underwent RALP between June 2012 and February 2015 at our institution. Patients with prior abdominal surgery were compared with those without prior surgery while considering the mean total operating, console, and port-insertion times; mean estimated blood loss; positive surgical margin rate; mean duration of catheterization; and rate of complications.

**Results:**

A total of 203 patients who underwent RALP during the study period were included in this study. In all, 65 patients (32%) had a prior history of abdominal surgery, whereas 138 patients (68%) had no prior history. The total operating, console, and port-insertion times were 328 and 308 (P=0.06), 252 and 242 (P=0.28), and 22 and 17 minutes (P=0.01), respectively, for patients with prior and no prior surgery. The estimated blood losses, positive surgical margin rates, mean durations of catheterization, and complication rates were 197 and 170 mL (P=0.29), 26.2% and 20.2% (P=0.32), 7.1 and 6.8 days (P=0.74), and 12.3% and 8.7% (P=0.42), respectively. Furthermore, whether prior abdominal surgery was performed above or below the umbilicus or whether single or multiple surgeries were performed did not further affect the perioperative outcomes.

**Conclusions:**

Our results suggest that RALP can be performed safely in patients with prior abdominal surgery, without increasing the risk of complications.

## INTRODUCTION

Radical prostatectomy is an effective treatment option for men with prostate cancer and currently offers the best long-term cancer control in patients with localized prostate cancer (1, 2). Recently, robotic-assisted laparoscopic radical prostatectomy (RALP) has become rapidly widespread and firmly established as a standard treatment choice for localized prostate cancer. Several studies have demonstrated that RALP has the advantages of decreased blood loss, lower perioperative complications, shorter length of hospital stay, and favorable oncologic outcomes (3, 4).

A previous report has demonstrated that prior abdominal surgery is a risk factor for increased surgical difficulty and complications during laparoscopic surgery (5). In fact, prior abdominal surgery has been recognized as one of the most important risk factors of the outcomes of laparoscopic surgery, owing to the increased risk of bowel injury caused by the needle and trocar insertions. However, little is known about the impact of prior abdominal surgery on the outcomes after RALP.

Therefore, the aim of this study was to evaluate the influence of prior abdominal surgery on the outcomes after RALP at our institution.

## MATERIALS AND METHODS

Between June 2012 and March 2015, 209 RALPs were performed at our institution. After the exclusion of 6 patients because of insufficient perioperative data, 203 patients were evaluated in this study. Demographic and perioperative data were collected prospectively. In addition, data regarding the final pathology, extracapsular extension, and margin status were also documented. A history of prior abdominal surgery was confirmed from a medical questionnaire administered at the first examination. Patients with no past surgical history were compared with those who had undergone at least one prior procedure. The surgical duration was documented using an operating time data sheet, which contained information regarding the total operating time, console time, port-insertion time, estimated blood loss, and prostate weight, as recorded by the operating room staff. Port-insertion time was defined as the time from the first port incision until the final port incision. Console time was defined as the time spent by the surgeon using the robotic console. For classification of the perioperative complications, the Clavien-Dindo grading system was used.

As a rule, the trocars are placed at least 8cm apart. The patients are placed in the Trendelenburg position, with their feet higher than their head by 25-30 degrees. The Hasson technique is typically performed above the umbilicus. After pneumoperitoneum is obtained, the initial adhesions are removed with laparoscopic scissors, typically placed through an 8mm robotic port. Deep pelvic adhesions are removed with robot assistance, after the robotic ports have been placed. The standard placement at our institution is a six-trocar system.

For the comparisons, the patients were divided into two groups: the prior abdominal surgery and no prior abdominal surgery groups. Patients with prior abdominal surgery were further classified into subgroups of single vs. multiple prior abdominal surgeries. Furthermore, the prior abdominal surgeries were also classified as above the umbilicus vs. below the umbilicus, regardless of whether the patient had also had prior abdominal surgery above the umbilicus.

Statistical analysis was down with JMP10 (SAS Institute, Cary, NC, USA) and EZR (Saitama Medical Center, Jichi Medical University, Saitama, Japan), which is a graphical user interface for R (The R Foundation for Statistical Computing, Vienna, Austria). Continuous data were expressed as the median and range, and were compared using the Mann-Whitney test. Categorical data were compared using the Fisher exact probability test or the chi-squared test. The survival curves were generated using the Kaplan-Meier method, and the difference in the survival curves were compared using the log-rank test. All P values of less than 0.05 were considered significant.

## RESULTS

Among the 203 patients, 65 patients (32.0%) had undergone prior abdominal surgery, while the remaining 138 patients (68.0%) had not. Of the 65 patients with prior abdominal surgery, 58 patients (89.2%) had only undergone one prior abdominal surgery, whereas 7 (10.8%) had a history of two prior abdominal surgeries. The types of prior abdominal surgeries are listed in Table-1. Of the 65 patients with prior abdominal surgery, 14 (21.5%) had surgery above the umbilicus only, while 51 (78.5%) had surgery either strictly below, or both above and below the umbilicus.

**Table 1:** Types of prior abdominal surgery (including overlap).

Appendectomy	26 (36.1%)
Inguinal herniorrhaphy	16 (22.2%)
Gastrectomy	7 (9.7%)
Cholecystectomy	4 (5.6%)
Nephrectomy	3 (4.2%)
Laparoscopic colectomy	3 (4.2%)
Laparoscopic cholecystectomy	2 (2.8%)
Laparoscopic partial-nephrectomy	2 (2.8%)
Colectomy	2 (2.8%)
Abdominal aortic aneurysm surgery	2 (2.8%)
Other procedures	5 (6.9%)
**Total**	**72 (100%)**

All data are presented as n (%).

The patient characteristics are listed in Table-2. There were no significant differences among the baseline patient characteristics in terms of age, body mass index, preoperative prostate-specific antigen level, pathologic stage, pathologic Gleason score, and rate of pelvic lymphadenectomy between the two groups.

**Table 2:** Comparison of patients with or without prior abdominal surgery.

Patient characteristics		Prior surgery	No prior surgery	P-value
No of patients (%)		65 (32)	138 (68)	
Mean age, years (SD)		68.3 (5.6)	67.6 (6.3)	0.55
Mean BMI, kg/m^2^ (SD)		23.9 (2.8)	23.6 (2.9)	0.37
Mean preoperative PSA, ng/mL (range)		10.5 (3.9-114)	10.1 (1.9-40)	0.41
Pathologic stage, n (%)				0.29
	pT2a	33 (50.8)	68 (49.3)	
	pT2b	0 (0)	1 (0.7)	
	pT2c	15 (23.1)	30 (21.7)	
	pT3a	16 (24.6)	25 (18.1)	
	pT3b	1 (1.5)	8 (5.8)	
Pathologic Gleason score (%)				0.62
	6	18 (28.1)	30 (22.4)	
	7	31 (48.4)	77 (57.5)	
	8	8 (12.5)	12 (9.0)	
	9-10	7 (10.9)	15 (11.2)	
Pelvic lymphadenectomy, n (%)		28 (43.1)	63 (45.7)	0.76

**SD** = standard deviation; **BMI** = body mass index; **PSA** = prostate-specific antigen

Table-3 shows the comparisons of the analyzed outcomes between the prior and no prior surgery groups. The mean total operating, port -insertion, and console times were 328 and 308 (P=0.06), 22 and 17 (P=0.01), and 252 and 242 minutes (P=0.28), respectively. The mean estimated blood losses, positive surgical margin rates, mean durations of catheterization, mean hospital stays were 197 and 170mL (P=0.29), 26.2% and 20.2% (P=0.32), 7.1 and 6.8 days (P=0.74), 15.3 and 14.6 days (P=0.30). The presence of complications was 8 (12.3%) and 12 (8.7%) in patients with and without prior abdominal surgery groups (P=0.42). With respect to the Clavien-Dindo grading system, less than 10% of the overall cohort experienced perioperative complications ≥ grade 2. Urinary tract infections and wound complication were the most common complications. No access-related complication occurred and there was no bowel or vascular injury. Furthermore, no patients required conversion to open prostatectomy and no deaths were encountered in this series.

**Table 3:** Comparison of patients with or without prior abdominal surgery.

Postoperative outcomes	Prior surgery	No prior surgery	P-value
Total operating time, min	328 (221-460)	308 (172-460)	0.06
Port insertion time, min	22 (5-76)	17 (7-90)	0.01
Console time, min	252 (142-402)	242 (125-407)	0.28
Estimated blood loss, mL	197 (5-800)	170 (5-750)	0.29
Positive surgical margin, n (%)	17 (26.2)	28 (20.2)	0.32
Duration of catheterization, days	7.1 (4-24)	6.8 (4-27)	0.74
Hospital stay, days	15.3 (9-36)	14.6 (10-33)	0.30
Perioperative complications, n (%)	8 (12.3)	12 (8.7)	0.42

Unless otherwise specified, the data are presented as the mean value (range).

**Figure 1:**
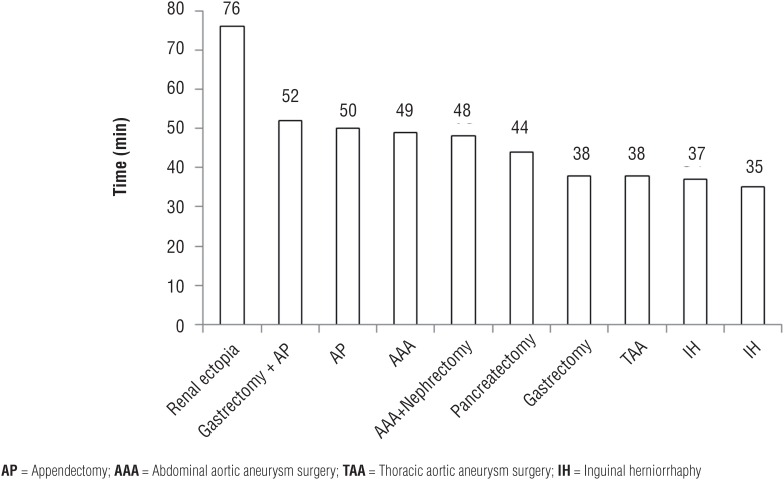
Time needed for port insertion according to different operative procedures.

Figure-1 shows the factors influencing the time needed for port insertion. In cases of prior open surgery, especially, the midline incision tended to require a longer port insertion time.

Table-4 shows the comparisons of the analyzed outcomes between patients with prior surgery above vs. below the umbilicus. Table-5 shows the comparisons of the analyzed outcomes between the single and multiple prior abdominal surgery groups. Whether prior abdominal surgery was performed above or below the umbilicus or whether single or multiple surgeries were performed did not further affect the perioperative outcomes.

**Table 4:** Comparison of patients with prior surgery below or above the umbilicus.

	Above the umbilicus	Below the umbilicus	P-value
	(n=14)	(n=51)	
Total operating time, min	322 (238-425)	330 (221-460)	0.73
Port insertion time, min	21 (10-44)	22 (5-76)	0.95
Console time, min	255 (167-369)	251 (142-402)	0.73
Estimated blood loss, mL	168 (5-800)	206 (5-700)	0.29
Perioperative complications, n (%)	3 (21.4)	5 (9.8)	0.35

Unless otherwise specified, the data are presented as the mean value (range).

**Table 5:** Comparison of patients with single or multiple prior abdominal surgeries.

	Single (n=58)	Multiple (n=7)	P-value
Total operating time, min	326 (221-460)	340 (238-441)	0.50
Port insertion time, min	22 (5-76)	26 (8-52)	0.55
Console time, min	252 (142-402)	251 (167-341)	0.92
Estimated blood loss, mL	195 (5-800)	211 (5-600)	0.91
Perioperative complications, n (%)	7 (12.3)	1 (12.5)	>0.99

Unless otherwise specified, the data are presented as the mean value (range).

## DISCUSSION

Patients with prior abdominal surgery may be at increased risk of complications, owing to the presence of intra-abdominal adhesions of the bowel to the abdominal wall, as well as the resulting distorted anatomy. Prior abdominal surgery was initially considered a relative contraindication to laparoscopic surgery (5). However, our operative results revealed that prior abdominal surgery was not associated with a significant increase in the total operating time, console time, positive surgical margin rate, and rate of complications, with only the port-insertion time showing a significant increase. Furthermore, a history of prior abdominal surgery below the umbilicus and more than one prior abdominal surgery were not significantly associated with a higher risk of complications.

Siddiqui et al. (6) reported that 27% of patients who underwent RALP were identified as having undergone prior abdominal surgery or inguinal hernia repair. Their study demonstrated that the adhesiolysis rate was much higher in those who had undergone prior abdominal surgery, at 24%, in comparison with 8% in those who had not undergone prior surgery. Overall, five bowel injuries were reported, with three related to the prior abdominal surgery. Many urologists intuitively associate prior abdominal surgery with increased complication rates and worse outcomes. In fact, there have been several reports of surgeons encountering severe fibrosis during radical retro-pubic prostatectomy in patients who have undergone inguinal hernia repair using mesh patches, leading to early termination of the procedure (7, 8). However, the increasing experience of surgeons and the improvements in the available technology have extended the indications for laparoscopic surgery and robotic surgery even to patients who have undergone prior abdominal surgery, with no adverse outcomes.

For example, previous reports have demonstrated that laparoscopic prostatectomy is possible after inguinal hernia repair and has little to no effect on the functional outcomes (9, 10). Moreover, recent reports have shown that RALP might not be contraindicated in patients who have undergone prior abdominal surgery or inguinal hernia repair using mesh patches (11). Ginzburg et al. (12) and Siddiqui et al. (6) reported that RALP could be performed safely and satisfactorily in patients with a history of a wide variety of prior abdominal surgery types. In these studies, prior abdominal surgery was not associated with differences in the overall operating time, robotic assist time, margin positive rate, or incidence of complications in patients undergoing RALP. Similarly, in the present study, RALP did not appear to be associated with any surgical difficulties, including an increased rate of pelvic lymphadenectomy, for patients with prior abdominal surgery and inguinal hernia repair using mesh patches (28/65 patients with prior abdominal surgery (43%) and 8/16 patients with inguinal hernia repair (50%) underwent lymph node dissection. Prior abdominal surgery was not associated with a significant increase in the total operating time, console time, positive surgical margin rate, and rate of complications, while the port-insertion time was significantly increased in the prior surgery group. When we investigate the factors influencing the time needed for port insertion, in cases of prior open surgery, especially, the midline incision tended to require a longer port insertion time. Furthermore, in cases of prior appendectomy developing into peritonitis, additional port insertions may be required, whereas in cases of inguinal herniorrhaphy, which cause mesh plug infection, a longer port insertion time than originally expected may be needed. Our results suggested that whether prior abdominal surgery was performed above or below the umbilicus or involved multiple surgeries did not generally affect the perioperative outcomes.

As summarized above, prior abdominal surgery did not worsen the outcomes of RALP, with only the port-insertion time being significantly increased. In terms of the rate of perioperative complications, no significant differences were observed in the prior abdominal surgery versus no prior abdominal surgery groups. Importantly, there was no case of bowel or vascular injury, no patients required conversion to open prostatectomy, and there were no deaths in this series.

To avoid the need for laparoscopic lysis of adhesions in a previously entered abdomen, the extraperitoneal approach may be considered. Madi et al. (13) demonstrated that the extraperitoneal approach offers similar clinical outcomes as the intraperitoneal approach, and the extraperitoneal approach avoids potential bowel injury or complications related to intraperitoneal urine leaks. Furthermore, Capello et al. (14) suggested that patients with prior abdominal surgery are best suited for the extraperitoneal approach because of the potentially lower risk of complications. Thus, the extraperitoneal approach may be an option in patients with prior abdominal surgery. However, while this approach may indeed be associated with a lower incidence of bowel injury and urine leakage into the abdominal cavity, it may not always be feasible if the surgeon is not experienced in this approach. In addition, the extraperitoneal approach is not suitable for surgeries such as extended lymphadenectomy. For these reasons, in the present study, the transperitoneal approach was safely performed, and achieved feasible outcomes without conversion to open surgery or increased perioperative morbidity for patients with prior abdominal surgery.

Our study has several limitations that should be considered when interpreting our results. First, the number of patients included in the current study was relatively small, and there may be a component of selection bias resulting from the retrospective nature of the study. Second, this study did not consider the learning curve of RALP. Secin et al. (15) reported that the learning curve for surgical margins after RALP plateaus at approximately 200 to 250 cases. Patients with prior abdominal surgery are often avoided in the surgeon's initial robotic-series; as the surgeons gain experience with the robotic procedure, the outcomes improve, and the willingness to take on more challenging cases is increased. However, despite these limitations, our data could serve as a reference for the assessment of outcome in patients with prior abdominal surgery after RALP.

## CONCLUSIONS

RALP appears to be a safe approach for patients with prior abdominal surgery. Prior abdominal surgery was not associated with significant increases in the total operating time, console time, positive surgical margin rate, and rate of complications, with only the port-insertion time showing a significant increase in this study.
